# Women’s Narratives about COVID-19, Preventive Practices and Sources of Information in Northwestern Tanzania

**DOI:** 10.3390/ijerph18105261

**Published:** 2021-05-15

**Authors:** Zaina Mchome, Gerry Mshana, Esther Peter, Diana Aloyce, Saidi Kapiga, Heidi Stöckl

**Affiliations:** 1 Mwanza Intervention Trials Unit, Mwanza P.O. Box 11936, Tanzania; zsmchome@gmail.com (Z.M.); esther.peter@mitu.or.tz (E.P.); diana.aloyce@mitu.or.tz (D.A.); Saidi.Kapiga@lshtm.ac.uk (S.K.); 2 Mwanza Centre, National Institute for Medical Research, Department of Sexual and Reproductive Health, Mwanza P.O. Box 1462, Tanzania; 3 Department of Infectious Disease Epidemiology, London School of Hygiene & Tropical Medicine, Keppel Street, London WC1E 7HT, UK; 4 Gender Violence and Health Centre, Department of Global Health and Development, London School of Hygiene & Tropical Medicine, 15–17 Tavistock Place, London WC1H 9SH, UK; Heidi.stoeckl@lshtm.ac.uk; 5 Medical Faculty, Ludwig-Maximilians-Universität München, 80331 Munich, Germany

**Keywords:** COVID-19, SARS-CoV-2, coronavirus, women, frame analysis, Tanzania

## Abstract

COVID-19 has affected millions of people across the world. We conducted a phone based qualitative study to explore women’s perceptions of COVID-19, knowledge of its symptoms, transmission, and prevention practices in Northwestern Tanzania. We also examined their sources of information about the disease. Findings show that much of women’s framing of etiology, symptoms, and transmission routes of severe acute respiratory syndrome coronavirus 2 (SARS-CoV-2) greatly reflects the World Health Organization (WHO)/Centers for Diseases Control and Prevention (CDC) frame. Their preventive practices against COVID-19 included the biomedical, cultural, and religious frames, as participants engaged traditional practices and spiritual interventions alongside public health recommendations. Mass media was the main source of information about COVID-19, and one of the trusted sources, in addition to religious and local leaders. To be effective, health promotion programs on pandemics should make more use of the mass media, and communal networks to reach populations.

## 1. Introduction

Coronavirus disease 2019 (COVID-19) is a respiratory disease caused by severe acute respiratory syndrome coronavirus 2 SARS-CoV-2 [1,2]. The World Health Organization (WHO) declared COVID-19 as a public health emergency of international concern in 30 January 2020 [3]. As of August 2020, developed countries reported twice as many cases as low- and middle- income countries, ascribed to lags in spread to low- and middle-income countries, and lack of testing capacity [4,5]. In Tanzania, the first case of COVID-19 was confirmed on 16 March 2020 [6]. To mitigate the outbreak of COVID-19, the Tanzanian government took a number of preventive measures, including extensive public health information campaigns, closure of schools and other educational institutions, banning large gatherings, restricting travel from affected countries, recommending hand washing and social distancing, quarantine of infected people, providing Personal Protective Equipment (PPE) for health care workers, and promoting wearing face masks [7]. Although Tanzania did not impose lockdowns, the government has continued to recommend prevention measures including handwashing, using sanitizers and locally made face masks, avoiding unnecessary international travel and going to large gatherings. Recently, the government has issued travel advisories and suspended flights to India to curb the spread of new strains of SARS-CoV-2. However, the effectiveness of these prevention and control strategies depends on knowledge and perceptions of the disease among the people in the community.

Since COVID-19 is a new disease, WHO and the United States Centers for Disease Control and Prevention (CDC) have been in the forefront providing information about its symptoms, modes of transmission, and possible preventive and curative measures. Based on the information provided by these institutions, people with COVID-19 report an array of symptoms that range from mild symptoms to severe illness. The most common symptoms are fever, dry cough, and tiredness. The less common symptoms include aches and pains, sore throat, diarrhea, conjunctivitis, headache, loss of taste or smell, a rash on skin, or discolouration of fingers or toes. And the serious symptoms are difficulty breathing or shortness of breath, chest pain or pressure, and loss of speech or movement [2]. COVID-19 is primarily transmitted through close contact with infected people via mouth and nose secretions (saliva, respiratory secretions or secretion droplets) released from the mouth or nose when an infected person coughs, sneezes, speaks or sings. It is also transmitted when a person touches contaminated objects or surfaces and then touches their eyes, nose or mouth. Other ways of transmission include close contact (within 1 m) with an infected person when those infectious droplets get into their mouth, nose or eyes, direct contact with infected people [2]. It is advised to keep a distance of at least 2 m from other people, washing hands with soap, wearing face masks when in public places, using hand sanitizers, avoiding contact with people who have fever or cough, covering the nose and mouth when sneezing/coughing, as well as avoiding unnecessary travel, going to crowded areas/large gatherings, and shaking hands [2]. In this paper we refer to this information on the symptoms, transmission, and prevention of COVID-19 as the “WHO/CDC frame.”

This study examined women’s understanding of COVID-19 in Mwanza, Tanzania and compared it with the WHO/CDC frame. The study sought to answer three questions. First, what do women know about symptoms and transmission of COVID-19? Second, what are the preventive measures that women are aware of and chose to engage in protecting themselves and their families against COVID-19? Third, how did they get the information about the COVID-19? Examining the knowledge and practices around COVID-19 among the general community would be helpful to provide better insight to address misconceptions about the disease and the development of preventive strategies and health promotion programs.

To our knowledge, no study conducted in Tanzania has explored how community members understand COVID-19, their preventive practices, and respective platforms where they get information about the disease. Understanding the community members’ narratives, practices, and sources of information about COVID-19 is an important step towards facilitating successful management of the pandemic.

We engage Ervin Goffman’s frame analysis theory [8], to examine and interpret women’s understanding COVID-19 in Tanzania. Goffman’s frame analysis theory is one of the approaches of studying and explaining how reality is socially constructed. A frame is a schema of interpretation. It is a collection of socially, culturally, or scientifically determined meanings of reality that individuals rely on to understand, explain, and respond to objects and events [8]. A frame, therefore, shapes how a phenomenon like COVID-19 is understood and communicated. As Goffman maintains, people interpret what is going on around their world through their primary framework—“one that is seen as rendering what would otherwise be a meaningless aspect of the scene into something that is meaningful” [8]. As Entman [9] emphasizes, frames select some aspects of a perceived reality and make them more salient in communicating text, in such a way as to promote a particular problem definition, causal interpretation, and/or treatment recommendation for the item described. The frame analysis theory helped to explain the women’s understanding of COVID-19 and compare it with the biomedical frame as laid out by WHO and CDC.

## 2. Materials and Methods

### Study Design and Methods

The study employed a qualitative approach to examine women’s understanding of COVID-19. Data was collected between July and September 2020. The recruitment procedures of the participants involved in this study has been described in detail elsewhere [10,11], but in summary, a total of 18 women aged 27 to 57 years were purposively selected to participate in qualitative telephone in-depth interviews. These women were selected from 85 women participating in the longitudinal mixed methods MAISHA study [12] who reported changes in their experiences of sexual intimate partner violence between the baseline and endline survey of the MAISHA trial. The MAISHA study investigates the predictors and consequences of intimate partner violence (ibid). Initially, these 18 women were involved in a qualitative research that sought to understand different forms of intimate partner violence from women’s own (*emic*) perspectives. After the outbreak of COVID-19 in Tanzania, the MAISHA investigators designed a low risk, nested phone-based study engaging the same 18 women to understand their knowledge/perceptions about COVID-19, and the effect of COVID-19 on the women, livelihood and violence.

Two trained female researchers interviewed women in Swahili, the widely spoken national language. The topic guide with open-ended questions and probes that covered various topics, including knowledge/perceptions about COVID-19, impacts of COVID-19 on women’s lives, impact of COVID-19 on their relationships/violence, and impact of COVID-19 on the community was used to solicit detailed information regarding women’s understanding of COVID-19 (see Annex 1, Supplementary Materials). The question guide was developed using the concepts from the scientific literature and the frame analysis theory. The duration of the interviews ranged from 31 to 86 min. The issues related to women’s understanding of COVID-19 were captured using questions included under the section on knowledge/perceptions about COVID-19. The topic guide was pilot tested to check the credibility of the questioning framework [13] and practicality of using phones for interviews. Participants were reimbursed a total of 8000 Tanzanian shillings via their mobile phones to compensate for their time.

Following the end of each in-depth interview, the audio files were transferred from the mobile smart phones to secured office computers before being analysed in Swahili using thematic approach. The interviewers (DA and EP) listened to the audios and wrote detailed interview reports which included detailed description of the findings. The content of these reports was guided by a predetermined standardized template that covered various aspects of the interview (see Annex 2, Supplementary Materials). To verify that the findings accurately represented the content of the audio files, the reports were reviewed by an independent qualitative researcher (first author)—who did not participate directly in data collection. The first author then read all the reports to identify the key emerging themes that reflected the women’s framing of COVID-19, which were further reviewed by the larger team involved in the analysis (GM, DA and EP). The identification of the emerging issues in the qualitative interview was informed by the predetermined topics that guided the telephone interviews and Goffman’s frame analysis theory [8].

The study was approved by the Ethics Committee of the London School of Hygiene and Tropical Medicine (Ref: 11918-3) and the Tanzanian Medical Research Coordination Committee (NIMR/HQ/R.8c/Vol. I/837).

## 3. Results

### 3.1. Women’s Understanding of COVID-19

#### 3.1.1. COVID-19 as a Deadly Disease

When asked about their understanding of coronavirus disease, most of the participants directly pointed to how they perceived COVID-19 in terms of its severe consequences, most notably, sudden and mass mortality. Remarks such as *ni ugonjwa mbaya* (‘is a bad disease’), *ni ugonjwa hatari* (‘dangerous disease’) *ni ugonjwa unaoua* (is a deadly disease’), were commonly used to describe COVID-19.

“This disease kills fast and quickly, it can kill a large number of people within a short period of time, it makes one not breath as normal and it affects the lungs and by affecting the lungs it causes death easily.” (1DI-16)

“As I understand, this coronavirus disease is very dangerous. It is a deadly disease.” (1DI-01)

“Corona is a bad disease that kills for a short period of time. It can kill many people in a short period of time.” (IDI-17)

Participants’ perception of COVID-19, however, evolved as time went by. In their accounts, most women spoke of COVID-19 as a “normal illness” or a disease like any other. Used in this context, the term “normal” does not refer to the high prevalence or to the grade of the illness, but expresses that such an illness is no longer new.

“From what I understand, this corona virus disease is a disease just like any other. We got the feeling that it was new because we had never seen it or heard of it but then after staying with it for several months, we have just started considering it as any other disease. Therefore, we have to protect ourselves like we do with other diseases.” (IDI-03)

#### 3.1.2. Perceived Etiology of COVID-19

Participants appeared to have a clear concept of the etiology of COVID-19, understanding that the disease is infectious, caused by a virus called corona. As one woman put it, “Coronavirus disease is a disease caused by a virus known as COVID-19.” (IDI-16)

#### 3.1.3. Perceived Symptoms of COVID-19

**Figure 1:**
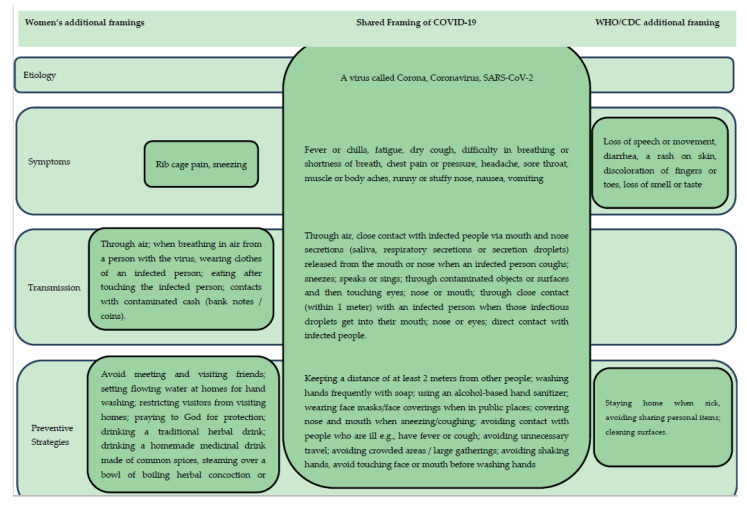
Women’s framing of COVID-19 vs. Biomedical frame.

In women narratives of COVID-19 symptoms, while severe headache, high fever, and dry cough were deemed to be mandatory, a broad range of other signs and symptoms were enumerated as manifestations of COVID-19. These include breathing difficulties, sore throat, chest pain, flu or cold, rib cage pain, body fatigue, muscle aches and crumps, sneezing, and gastrointestinal symptoms including nausea and excessive vomiting (see Figure 1).

“The symptoms of corona virus disease include severe headache, having high fever, sore throat, difficult breathing, and one may fail to breath and sometimes vomits a lot. I think those are the main signs […] including body weakness.” (ID-I1)

“The symptoms of this coronavirus disease are coughing, high fever, sore throat, rib cage pain, chest pain and severe headache.” (IDI-10)

“We were told that the symptoms of the disease include rib cage pain, severe headache, sneezing, and others.” (IDI-06)

The prevailing schemas about COVID-19 signs and symptoms influenced participants’ interpretation and response to symptoms of other illnesses—such as malaria and flu—during this period. Some narratives pointed to a common use of the prevailing constructs about COVID-19 signs and symptoms in interpreting and responding to symptoms of other illnesses, which include malaria and flu, during this period. For instance, some women reported to have experienced some episodes of symptoms related to those of coronavirus disease such as fever, coughing and flu during this period, and associated them with COVID-19. As one participant put it, *“Most* *people when they had flu, they associated it with corona.”* (IDI-07). While some of them went to the hospital for check-up and found that they had no corona virus disease, others resorted to self-care—using both pharmaceutical drugs and traditional remedies to rectify their conditions.

“When corona was announced, there was a time when I felt sick. I had high fever, flu, and I felt very cold. I think you know it is the seasonal period when people get sick often. So, I thought to myself may be this is corona because the flu got really serious, so I took malaria and flu medicine and I later felt fine. You just find yourself worried and taking other precautions, as for me, I decided to stay indoors and even used the traditional medicines and remedies for my own sake. The sickness lasted for a week and I was back to being normal.” (IDI-16)

#### 3.1.4. Perceived Transmission Routes of COVID-19

The women’s knowledge about pathways through which coronavirus disease could be transmitted overlaps with the biomedical frame of the disease, although the two are not entirely congruent. The majority affirmed that the virus that causes COVID-19 spread from human-to-human, mainly through respiratory droplets from the nose or mouth produced when a person with COVID-19 is talking, coughing, sneezing or blowing their nose.

“Corona is a disease that is transmitted through the respiratory system. When someone sneezes without wearing *barakoa* (face mask/face covering) or blocking the propelled droplets with a hand, when the watery substances reach another person, it is one of the sources through which one could be infected with coronavirus.” (1DI-01)

“Coronavirus disease spreads through the nose and mouth when a person splits watery substance. […] When the infected person sneezes and he is near you, the watery substances coming from their mouth or nose if it gets to you, it’s likely for you to be affected by the disease.” (IDI-16)

The participants’ framing of transmission routes of COVID-19 was also influenced by the widely held schema regarding impurities carried by bodily substances from infected person. The majority believed that body contacts with an infected person, and contact with contaminated hands, and then touching eyes, nose or mouth are the main ways one could catch COVID-19. Some also held that one could be exposed to the infected droplets through wearing clothes of an infected person, and eating after touching the infected person. Similarly, some participants believed that contact with contaminated surfaces, objects, and cash (bank notes/coins) particularly if exchanged in hotspots such as shops and public transport can also expose someone to coronavirus.

“If you touch a person with corona you get the disease […] another way is if you eat after touching the infected person you can easily get the disease. And also, if you wear clothes of the infected person you can also get the disease, and the final one is through money exchange like changes from the shop, public transports and others, if the money has been touched with an infected person and you also touch it, you can easily get corona, I think those are the ways of being infected with coronavirus.” (IDI-04)

“We were told that one might get the disease by holding each other’s hands, hugging each other, touching eyes, nose or mouth.” (IDI-06)

“Someone can also get the disease if they touch a person with the disease without any protection. You might touch her/him and get in contact with those viruses.” (IDI-15)

Interestingly, most of participants during in-depth interviews felt that COVID-19 is transmitted through air, a conception that may have been reflective of the current COVID-19 information landscape in Tanzania. When echoing on this, participants consistently referred to what they were told or taught by others.

“They taught us that one can even get it (COVID-19) through air. When you stay close to someone with the corona virus disease, they spread it to you through air [...] that’s what I was taught.” (IDI-12)

“Corona virus disease is the disease that spreads through air [...] if someone breathes in air from a person with the coronavirus disease [...] that’s how you can get the disease.” (IDI-10)

“First of all, I know that this is a disease that spreads through air and other things like those. That’s how I understand. I don’t know about other things. I know it spreads through air and sitting or staying close to someone else.” (IDI-09)

“It’s a dangerous disease that is transmitted through air or watery substance like saliva.” (IDI-7)

“What I know about corona is that schools and even some businesses were closed because of it, this was because the disease spreads through air.” (IDI-16)

### 3.2. Preventive Practices against COVID-19

Although participants’ theoretical knowledge of precautions against COVID-19 mainly reflected biomedical concepts (see Figure 1), their daily preventive practices regarding the disease were embedded in multiple frames, including the WHO/CDC frame, traditional medicine, and religious frames.

#### 3.2.1. Preventive Practices Based on WHO/CDC or Biomedical Frame

Most of the participants’ knowledge of precautions against COVID-19 overlapped to a large extent with the biomedical concepts, and the majority used them in their daily practices.

Their narratives suggest that they protected themselves and their families against COVID-19 through engaging in frequent hand washing with running water (*maji tiririka)* and soap, used hand sanitizers, installing flowing water at the entrance of their homes for people to wash hands before entering their homes, restricting visitors from visiting their homes, worn *barakoa* (referring to face masks/face coverings) each time particularly when they went in public places, avoided crowded place/large gatherings, meeting and visiting friends, and shaking hands while greeting people as instructed by the government.

“What I actually did was avoiding large gatherings. When I went out, I made sure I have my *barakoa* on. I also stopped going to the bar or meeting up with friends.” (IDI-16)

“Was by washing hands more often, using sanitizers when am back home or even going out even in public transportation I really had to wear *barakoa* but also in places which are very crowded.” (IDI-17)

Others also added that they put water at the home entrance for people to wash hands before entering their homes, restricted themselves and visitors from visiting each other’ homes, and avoided shaking hands while greeting people. Measures were taken to protect children and the elderly from catching SARS-CoV-2. For example, parents limited their children’s movements outside home, stopped them from seeing friends, visiting neighbours or going to shops for groceries. For the elderly, given their low immunity, they restricted them from unnecessary travels and meetings.

“I prepared a bucket full of water and a liquid hand washing soap. So, whenever I received a visitor or when my children came from school, they had to wash their hands first before coming in.” (IDI-09)

“You know I even stopped entertaining visitors at home, we were told not to invite people at our homes, you can invite a person but you don’t know if they have corona or not.” (IDI-13)

“Whenever my kids wanted to go to church it was a must they go with their masks on. We placed a cane full of water and soap so that the children would wash their hands whenever they came back from anywhere. So, we protected ourselves in that design.” (IDI-15)

#### 3.2.2. Preventive Practices Based on the Traditional Medicine Frame

The preventive practices against COVID-19 also had implicit roots in traditional medicine, as alongside with biomedical and religious measures, some women also used traditional preventive measures to protect themselves and their families. These have been described in detail elsewhere [10]. These include (1) drinking a homemade medicinal drink made of common spices such as ginger, garlic, cinnamon, clover, lemon, and leaves of plants such as lemongrass, eucalyptus, lemon, neem, guava, and *Kashwagara* (local mint plant); and (2) steaming over a bowl of boiling herbal concoction or spices and inhale steam coming out while covered up with a cloth. To participants, everyone in the family, including young children, was involved in the steaming, except the sick and the elderly.

“I have steamed myself a lot, my kids as well have done that, […] what I know about the steaming process is that you really sweat. The ingredients that I used were eucalyptus, guava leaves and other herbs, we boiled them, then steamed ourselves while covering with a sheet that doesn’t allow air to penetrate, we all did this, even my husband, after the whole process we felt okay, you feel like you’re no longer tired.” (IDI-#02)

In this context, mistrust on the biomedical precautions was not the motive behind women’s use of traditional remedies, rather, the women’s need to maximize their protection given the widely held schema that the disease has no cure.

“We decided to steam ourselves because we heard people saying that the disease has no cure so we should take all the necessary precautions and even steam ourselves.” (IDI-#02)

#### 3.2.3. Preventive Practices Based on Religious Frame

The religious beliefs appeared to have permeated the participants’ meaning systems and interpretations of COVID-19, and thus informed their actions. The nature of the coronavirus and the unfeasibility of some of the recommended biomedical preventive measures in the local context made most of participants, regardless of their religious affiliation, feel helpless, and thus considered God’s mercy as their only hope. Based on this feeling, most participants resorted to spiritual intervention for their protection against COVID-19.

“I also prayed to God that this disease should end. I just prayed to God for protection.” (IDI-08)

Those who indicated to have prayed about COVID-19 seemed to believe that God has answered their prayers, with some participants believing that the disease no longer exists in the country, which influenced their decision to not adhere strictly to precautions as they did before.

“We prayed and reminded God to protect Tanzania from the coronavirus disease and he listened. The situation is as you can see right now. We don’t wear *barakoa* like before. We only wash our hands with flowing water, in the past we stopped gathering but now we can attend our gatherings as usual.” (IDI-01)

### 3.3. Sources of Information about COVID-19

Participants received information on coronavirus disease from multiple sources, with the majority indicating that they heard about it through mass media, especially radio and television. Only few participants mentioned newspapers as the source where they firstly heard about COVID-19.

“The first time I heard of it was on radio and TV. They said there was an outbreak of a dangerous disease called corona. They announced that we had to be very careful.” (IDI-06)

“I got information about the disease from the radio […] I heard from the Television that there is a disease called corona which started in China, but later spread to other countries then reached Kenya and finally in Tanzania. I got news about the disease before it even reached in our country through the Media.” (IDI-16)

In addition to mass media, women also received information about COVID-19 through social media such as YouTube, WhatsApp, and Facebook.

“I got more information from the Television but also radios and phone. I got information from the social media like Facebook and WhatsApp.” (IDI-10)

“I really like watching You Tube videos, I first saw news about the disease from there, they said it killed many people in China, so from there I started making follow ups on what was going on until it reached Tanzania. What I can say is YouTube was my source of information, or generally the internet gave me information.” (IDI-04)

Religious leaders emerged as one of the predominant conveyors of information about COVID-19 to people in the community. The majority participants mentioned to have heard about COVID-19 at church/mosque through their religious leaders, with some elucidating that they only took it serious after their religious leaders talked about it.

“I got news through the radio and also in church, but for church they really emphasized about it, they made us become really aware of the disease existence […] so that made us realize that the disease is serious. Although they talked about it in the TV, we came to take it serious when we heard from the church.” (IDI-07)

“I also got the information from the religious sources. They announced this (about COVID-19) for us in mosques.” (IDI-05)

“I heard from the television but also the church really talked about it, and it’s when I realized that this was serious.” (IDI-17)

Women’s social networks emerged as another important source of COVID-19 information, through daily conversations with relatives, friends, neighbors, co-workers, and fellow women during their routine microfinance group meetings. Other participants echoed on the role played by street/local leaders, public announcements by different actors including the government, and people with leadership positions at different work places.

“From the beginning I got information from people around me, my neighbors, workmates and business partners, in our daily conversations we would talk about the increase of patients confirmed having COVID-19 by the government and so on.” (IDI-16)

“I got the information from people who were going around announcing about the disease and teaching us how to protect ourselves.” (IDI-12)

“I heard about the disease through community sensitization which was done by the government through the local government and other government officials who were selected to provide us information on coronavirus. They gave us education on precaution to be taken like washing of hands, using of masks and others.” (IDI-11)

“We were also informed in the small supportive groups we attend. We formed these groups to support one another (financially) but we had to close them to avoid gatherings.” (IDI-06)

In general, although participants received information about COVID-19 from multiple sources, they reported to only trust the mass media, religious and local leaders, with some articulating their mistrust on news from individual people and social media. The long-term reliability of news from the mass media and religious/local leaders seemed to have influenced the participants trust on them, unlike their experience with social media which they depicted as producing rumors and misinformation instilling fear to the public.

“I liked listening to news which I heard from the government through our local leaders, or the media, they gave us trusted news on the spread of the disease and the precautions to be taken. I really didn’t believe much on the news from the social media because people gave rumors that brought fear to us, so I had to avoid such news.” (IDI-11)

“I trusted what was broadcasted in the radio, and you know when you listen to people, they would give you false information, but the media gives you trusted news/information.” (IDI-04)

“I got information from the media like the television and radios […]. I couldn’t consider information I heard from other sources like people. I only trusted the media.” (IDI-17)

## 4. Discussion

The findings suggest that much of the women’s framing of etiology, symptoms, and transmission routes of SARS-CoV-2 resonates with the biomedical framing of the illness. This is consistent with other studies in Africa [14], and Asia [15,16,17] which indicated high levels of COVID-19 knowledge among the general population. The women’s use of biomedical concepts in interpreting the etiology, symptoms, and transmission routes of SARS-CoV-2 was expected as the general public in Tanzania has been exposed to extensive health education, by the Ministry of Health in collaboration with other public health actors, on COVID-19 since the disease emerged.

Although participants in a study conducted in Asia reported that COVID-19 virus is not an airborne disease [17], women in this study believed that COVID-19 virus was transmitted through air. This finding indicates that women had a proper biomedical knowledge regarding modes of SARS-CoV-2 transmission consistent with the amounting scientific evidence which shows that COVID-19 is an airborne disease [18]. Based on our study findings, health authorities would benefit from utilizing both mass media and community networks, particularly religious leaders in disseminating these messages. As other studies have shown, religious leaders are important vehicles in promoting health behavior, particularly when they are equipped with proper knowledge [19].

Participants’ use of schemas about COVID-19 signs and symptoms in interpreting and responding to symptoms of febrile and other respiratory illnesses—such as malaria and flu—should be taken cautiously, as it fuels self-medication practices, which have many adverse health effects including delayed treatment-seeking [20]. People’s tendency of confusing the symptoms of COVID-19 with similar symptoms of other illnesses is also reported by previous studies elsewhere in Africa [14]. We recommend that the general public should be informed about the key differences between COVID-19 and other respiratory diseases as documented by the leading public health institutions such WHO and CDC [21,22].

Women’s narratives indicated that people turned to spiritual intervention for their protection against COVID-19. This response is not surprising as the evidence show that in times of crisis, people tend to resort to religion for comfort and explanation [23]. The influence of the religious beliefs in shaping women’s interpretations of and practices around COVID-19, and the normalization of COVID-19 revealed in this study needs attention, as it motivates people’s poor adherence to the recommended precautions against COVID-19. The ministry of health needs to keep educating the community that the disease still exists thus they should remain vigilant and take the recommended precautions. Mass media, community leaders and religious leaders would be helpful in challenging harmful beliefs around COVID-19 as they are trusted by the community members.

Women in this study executed the biomedical knowledge they had on precautions against COVID-19 in their daily lives, as the majority reported taking precautions such as wearing face masks/face coverings, avoiding crowded places and practicing proper hand hygiene. This indicates a general willingness of participants and the public at large to make behavioral changes in the context of COVID-19 pandemic. However, the analysis revealed the existence of medical pluralism [24] in the context of COVID-19, as women draw on biomedical and non-biomedical systems to protect themselves from catching SARS-CoV-2 [10]. As Muela [25] states, a society where medical pluralism is well established is rich in knowledge stemming from different explanatory systems. For instance, preventive practices against catching SARS-CoV-2 that women explained were influenced by multiple frames including biomedical, religious, and cultural frames. In this context, turning to multiple prevention measures was not because of people’s poor knowledge or mistrust of the biomedical measures. Instead, this was due to the novelty of the coronavirus and the uncertainties it caused including the lack of its cure and the rate at which it caused fatality. Thus, people may turn to every measure perceived could be preventive against the virus. However, the finding that children were also involved in steam inhalation with herbal concoction as a prevention of COVID-19 needs attention, given its possible adverse effects to children’s health [26].

This study has several strengths and weaknesses. Despite that COVID-19 is a pandemic that has affected and killed millions of people across the world, including Tanzania, this is the first study in the country to explore how community members—women in particular—frame the disease and navigate through it. Additionally, conducting this study using Swahili—the local language—and analyzing the data in the original language resulted in rich data. Our study is also unique in that, given the current context; it engaged telephone interviews to collect information from the participants, which enhanced safety to both participants and interviewers. Because the study was conducted in an urban setting of the country, and the number of participants was small, the generalizability of the findings may be limited. However, we believe the study’s findings are highly relevant to the wider Tanzanian context as the women involved were recruited from the general community and had diverse socio-demographic characteristics. Additionally, although interviewing the participants over the phone permitted more anonymity and privacy, thus making women relaxed, willing to talk freely and disclose intimate information; it limited the possibility of capturing nonverbal and contextual data, which are important in enhancing the interpretation of participants’ verbal responses [27,28]. To enhance the quality of our findings, we used different ways to compensate for the absence of nonverbal responses, including paying attention to intonation [29], hesitations and sighs [30]. Lastly, to ensure in-depth discussion, and at the same time avoid participant fatigue, using this approach compelled us to keep the interviews short compared to how it would be if we would have used face-to-face interviews.

The findings confirm that mass media, religious leaders, and local leaders were the most trusted sources regarding COVID-19. There is a need to examine (1) these opinion leaders’ knowledge of COVID-19, and (2) reasons why they were trusted more than other sources. This knowledge could help identify the knowledge gaps regarding COVID-19 among the opinion leaders, thus enable health programmers to develop specific interventions focused on dispelling the myths, and identify ways to engage the local and religious leaders in promoting healthy life in the community. Additionally, as also recommended by Mshana et al. [10], there is a need of studies involving larger sample sizes to examine the community members’ knowledge and perceptions of COVID-19 in this population or other similar contexts.

## 5. Conclusions

Through the use of frame analysis, the findings show that biomedical conception of COVID-19 is the primary framework used by women in assigning meaning to the etiology, symptoms, and transmission of the disease—the knowledge that they mainly received through mass media, religious institutions, social groups and local leaders. The analysis also revealed the existence of medical pluralism in the context of COVID-19—as people engaged biomedical and traditional systems to protect themselves from catching SARS-CoV-2. Our findings provide useful insights in designing interventions to reduce the COVID-19 transmission as well as related future pandemics. The women in this study trusted information about COVID-19 from mass media, religious leaders and local leaders. Based on our findings, public health responses during pandemics should use mass media and community networks to deliver messages about symptoms, etiology, prevention and treatment of a particular disease. However, further research is needed to determine how these community-based sources of information can be successfully integrated into preventive and health promotion interventions. From the findings, addressing existing misconceptions around COVID-19 is key for the successful preventive efforts of the pandemic.

## Data Availability

The data presented in this study are available on request from the corresponding author, and after receiving permission from Tanzanian Medical Research Coordination Committee.

## Supplementary Materials

The following are available online at https://www.mdpi.com/article/10.3390/ijerph18105261/s1, File S1: In-depth interview question guide, File S2: Report writing template.
