# Supplementary material for: Women’s Narratives about COVID-19, Preventive Practices and Sources of Information in Northwestern Tanzania
Source: Int J Environ Res Public Health. 2021 May 15;18(10):5261. doi: 10.3390/ijerph18105261 (PMC8156351; doi:10.3390/ijerph18105261)
Supplement: Supplementary file 1 [file ijerph-18-05261-s001.zip › S1_In-depth interview question guide.pdf]

The effect of COVID-19 on Women, Livelihood and Violence in Mwanza, Tanzania

IN-DEPTH INTERVIEW GUIDE

1. Knowledge/Perceptions about COVID-19

- What do you understand of Corona virus disease/Corona disease?
- What do you know about the disease?
- How did you hear about it? **Probe:** If from person, ***who was it? Where?***
- What are the symptoms of the disease?
- How can someone get the disease?
- How is it treated? **Probe about:**
    - Biomedical/hospital treatment
    - Traditional medicine. **Probe:** on specific practices
    - Other ways of treating it
- From what you have just described, what is the most effective way of treating the disease? **Probe:** Why do you think so?
- Is there a way one could protect themselves from getting it? **Probe:** How?
- From what main source do you get information about the disease? Probe: about sources such as people, media, religious sources.

2. Impact on their lives

- How has the disease affected you and your family? **Probe about**:
    - Effect on her
    - Effect on their children
    - Effect on their relationship with husband/partner
- How has the disease affected your income generation activities? **Probe:**
    - What effect do you see on your business?
    - If any effect, why is that?
    - Effect on income generation activities of partner
- How has it affected your health? **Probe:**
    - About dealing with the symptoms
    - About feeling depressed, sad or being worried
- What steps have you taken to protect yourself from getting the disease?
- What have you done to protect your family from getting the disease? **Probe about:**
    - Protecting their children
    - Protecting elderly
    - Protecting other people in their household e.g. relatives, house helpers, gardeners
- Did you get the disease or suspect that you got it? **If yes, probe:**
    - How did you know that it was Corona virus disease/Corona disease? Or why do you think it was corona?
    - What did you do? **Probe:**
        - For different actions, for example, went to health facilities, consulted traditional healers, used traditional herbs
        - Ask about what motivated her decisions

- What treatment did you receive?
- For how long were you affected?

- Did anyone else in your family get the disease? **If yes, probe:**
  - Who was it?
  - What did you do? **Probe:**
    - What treatment did they receive?
    - Where?
    - For how long?
    - Ask about what motivated their decisions
- How did getting the disease affect your family life?
  - How did it affect your relationship with your children?
  - How did it affect your relationship with your husband/partner?
  - How did it affect your relationship with other community members? **Probe:**
    - Did you receive assistance from them? **Probe:**What kind of assistance?
    - Did they keep away from you and your family**? Probe:**
      - How did it happen?
      - How did you feel?

3. Relationship/Violence

- **Since the disease started:**
  - Has your relationship and your joint life changed since the disease started? **Probe:**
    - Are you spending more time at home with your partner?
    - Are you meeting less friends?
    - Are your children at home?
  - Have you argued more with your partner? **Probe:** what have you mostly argued about?
  - Has your partner become more easily irritated than in the past? **Probe:** why?
  - Has your partner become violent towards you? **Probe:**
    - In what specific ways? Can you give specific examples?
    - Why?
    - Is this more than before?
  - Does your partner take alcohol? **If yes**, has your partner increased or decreased their use of alcohol? **Probe:**
    - Why?

- **Since the disease started:**
  - How did your children spend most of the time when schools were closed?
  - Were you worried about your children not being in school? **Probe**: Why?
  - Is your partner spending more time with his family? **Probe:**
    - How does this affect your relationship?
    - Did your partner spend more time with family when schools were closed?
    - Have your children irritate your husband/partner more easily?

- Have your children irritate you or your partner more easily? Have you or your partner hit your children more frequently than in the past? **Probe:**
    - Why?
    - Probe for other forms of violence e.g. yelling / verbal insults

4. Impact on community

- How do people in your community understand the disease?
- How has the disease affected your community?
- What precautions are people taking in your community against the Corona virus disease/Corona disease? **If not mentioned, probe** on traditional preventive measures.
- How has the disease affected income generation activities in your community?
- If anyone in the community has corona or thinks they have it, how does the community react? **Probe about:**
    - Support and type of assistance
    - Keeping distance and social isolation
- How do people in your community talk about the Corona virus disease/Corona disease? **Probe:**
    - Are there rumours in your community around the disease? **If yes:** what are they?

THANK YOU FOR YOUR TIME.
