# Supplementary material for: Women’s Narratives about COVID-19, Preventive Practices and Sources of Information in Northwestern Tanzania
Source: Int J Environ Res Public Health. 2021 May 15;18(10):5261. doi: 10.3390/ijerph18105261 (PMC8156351; doi:10.3390/ijerph18105261)
Supplement: Supplementary file 1 [file ijerph-18-05261-s001.zip › S2_Report writing template.pdf]

COVID-19 QUALITATIVE STUDY

IN-DEPTH INTERVIEW REPORT TEMPLATE

Objectives

- To set a standardized format for writing the IDI reports after the completion of each phone interview.

Sections of the report

1) DATA COLLECTION PROCESS
   - 1.1 Time & date of the interview
   - 1.2 Length of the interview
   - 1.3 How the respondent was reached
     - 1.3.1 No of calls/ attempts
   - 1.4 Consenting process
     - 1.4.1 Participant's reaction on the invitation for the phone interview
     - 1.4.2 How the consenting process went
     - 1.4.3 Any questions asked during the consent women wanted clarification for
   - 1.5 Interview venue
     - 1.5.1 Interviewer's venue
     - 1.5.2 Respondent's venue
   - 1.6 How the interview went
     - 1.6.1 Participant's participation
       - ❖ Activeness of the participant
       - ❖ Ability to understand questions
       - ❖ Any barriers for the participant's participation
       - ❖ Interruptions
       - ❖ People overhearing
       - ❖ What went wrong and if addressed, how
     - 1.6.2 Interviewers impression of the interview eg. Good, challenging, difficult, why...

2) FINDINGS
   - o Participant's demographics
   - 2.1.1 Age
   - 2.1.2 Occupation
   - 2.1.3 Ethnic group
   - 2.1.4 Religion
   - 2.1.5 Marital status
   - 2.1.6 Education
   - o Summary of the findings

Note: The summary of the findings will be descriptive and written following the themes in the guide.

3) REFLECTIONS ON HOW THE METHOD WORKED

3.1 How the technology worked / Technical aspects of the phone interviews

3.1.1 Connection clarity
3.1.2 Audibility
3.1.3 Interview recordings
3.1.4 Calls being cut/ hanged up
3.1.5 Battery issues

3.2 Respondents happiness and distress

3.2.1 Any indications coming up?

3.2.2 Notable stories

3.2.3 Unsureness on your side and why?

3.2.4. Ethical concerns

3.3 Interviewer's reflection of how the method worked

3.2.1 Thoughts on how the method worked well
3.2.3 Thoughts on how the method did not work well

4) CONCLUSIONS AND RECOMENDATIONS

4.1 About the method
4.2 About the findings
